# Protein Aggregates and Aggrephagy in Myopathies

**DOI:** 10.3390/ijms24098456

**Published:** 2023-05-08

**Authors:** Sara Gibertini, Alessandra Ruggieri, Marta Cheli, Lorenzo Maggi

**Affiliations:** Neuroimmunology and Neuromuscular Diseases Unit, Fondazione IRCCS Istituto Neurologico “Carlo Besta”, 20133 Milan, Italy; sara.gibertini@istituto-besta.it (S.G.); marta.cheli@istituto-besta.it (M.C.); lorenzo.maggi@istituto-besta.it (L.M.)

**Keywords:** protein quality control (PQC), protein aggregates, aggresome, aggrephagy, muscle disorders

## Abstract

A number of muscular disorders are hallmarked by the aggregation of misfolded proteins within muscle fibers. A specialized form of macroautophagy, termed aggrephagy, is designated to remove and degrade protein aggregates. This review aims to summarize what has been studied so far about the direct involvement of aggrephagy and the activation of the key players, among others, p62, NBR1, Alfy, Tollip, Optineurin, TAX1BP1 and CCT2 in muscular diseases. In the first part of the review, we describe the aggrephagy pathway with the involved proteins; then, we illustrate the muscular disorder histologically characterized by protein aggregates, highlighting the role of aggrephagy pathway abnormalities in these muscular disorders.

## 1. Introduction

The accumulation of misfolded proteins is a common pathological characteristic of degenerative diseases of the central nervous system, but it is also present in muscular disorders where the inhibition of autophagy leads to protein aggregates and/or vacuoles formation, causing muscle fiber degeneration and myopathy [1,2,3].

Autophagic vacuolar myopathies (AVMs) and protein aggregate myopathies (PAMs) represent a series of clinically heterogeneous myopathies characterized by lysosomal or extralysosomal accumulation of proteins or other substances, with varied ages of onset, progressive disease course and variable degree of severity [4,5,6].

AVMs, characterized by the presence of autophagic vacuoles, can be classified into three groups based on specific pathomechanical and morphological findings.

Specifically, the first group is characterized by the deficiency of the lysosomal α-1,4-glucosidase enzyme, causing Pompe disease, and the second by abnormal and functionally impaired lysosomes as in X-linked myopathy with excessive autophagy (XMEA) and Danon disease. The third group is defined by secondary lysosomal dysfunction leading to rimmed vacuoles, popcorn-like clear membrane-bound organelles with a densely blue rim by hematoxylin and eosin staining. This group comprises inclusion body myopathy with Paget disease and frontotemporal dementia (IBMPFD), hereditary and sporadic inclusion body myopathy (hIBM and sIBM) and oculopharyngeal muscular dystrophy (OPMD), among others [4,7]. Recently, a new AVM caused by mutations in the *PLIN4* gene has been described [8].

PAMs are mainly identified by the presence of extralysosomal protein aggregates, and it has been hypothesized that abnormal protein synthesis, impaired extralysosomal degradation system or the integration of proteins in the intracellular structured constituents could contribute to the development of protein aggregation [5,6,9,10,11]. One type of PAMs could be classified as “catabolic”, where the aggregation is likely due to extralysosomal protein degradation failure, as observed in but not limited to myofibrillar myopathies (MFMs), cores diseases, sIBM and hIBM, reducing body myopathy and oculopharyngeal muscular dystrophy. In another subgroup, defined as “anabolic PAM” marked by actin filament and granular myosin aggregates, the accumulation is ascribable to synthetic/developmental defects of actin and myosin filaments, notably associated with onset often in early childhood. Nemaline myopathies and myosinopathies belong to this group [5,6,9,12,13].

Both AVMs and PAMs show some overlap due to similarities among the pathological mechanisms.

To date, no treatment is available for any of these disorders, except for the enzyme replacement therapy in Pompe disease [14,15].

This review focuses on aggrephagy, a specialized form of autophagy responsible for the removal of protein aggregates, a mechanism that may be relevant in specific muscle diseases and potentially representing a therapeutic target. Despite this, so far, only a few studies investigating aggrephagy in muscle diseases have been reported in the literature.

## 2. Protein Quality Control (PQC) System: Protein Misfolding and Aggrephagy

### 2.1. Protein Folding and Misfolding and Molecular Helpers

The folding of newly synthesized proteins is a highly demanding process, especially in the crowded cellular environment where protein content might reach concentrations of 300–400 g/L [16]. Folding is ruled by kinetic (the vectorial nature of translation) and thermodynamic (the need for energy minimization) factors. It can occur co-translationally, during ribosomal-assisted synthesis either on free ribosomes or on ribosomes that are bound to the sarcoplasmic/endoplasmic reticulum (SR/ER) [17], as well as post-translationally in the cytoplasm or in confined organelles (mitochondria and endoplasmic reticulum) [18].

The newly synthesized polypeptides, while reaching their native structures, fluctuate between unfolded and folded states, depicting a funnel-shaped energy landscape [19]. Moreover, while the energy landscape is smoother for small proteins (<100 amino acids in length) that can fold very rapidly within microseconds, larger proteins (>100 amino acids), which account for ~90% of the proteome in a cell, have a rougher path because of a higher tendency to collapse in the aqueous solvent [16,19]. Likewise, proteins might be intrinsically disordered within the cytoplasm, reaching a tri-dimensional state only upon binding with membrane surfaces or macromolecules [16,20,21]. In addition, this complex situation is further complicated by cellular unpredictable events such as genetic mutations, errors in transcription or translation and chemical or temperature stresses in the cellular environment.

It is no surprise then that proteostasis (the maintenance of protein homeostasis) involves numerous pathways composed of hundreds of proteins such as specialized chaperones and folding enzymes, facilitating folding and refolding, and degradation components like the ubiquitin-proteasome system (UPS) and autophagy for the removal of the permanently misfolded and aggregated proteins [16,22,23,24].

In human cells, over 300 chaperones and co-chaperones [25] are known to assist in de-novo folding, refolding of denatured proteins, assembly of protein complexes and protein degradation [26].

Heat shock proteins are a special family of chaperones that are upregulated upon thermal stress or other proteotoxic stresses that elevate the concentrations of aggregation-prone intermediates. They are classified based on their molecular weight (HSP40, HSP60, HSP70, HSP90, HSP100 and the small HSPs) [27].

The HSP70 family includes 47 proteins encoded by 17 genes in the human genome that can be stress-inducible, such as HSP70s, or constitutively expressed as the heat shock cognate HSC70 [28].

All HSP70 members share common structural features, having a 45 kDa N-terminal ATPase nucleotide binding domain (NTD) and a 25 kDa C-terminal substrate binding domain (SBD) that is able to recognize co-chaperones through its conserved motif EEVD [29,30,31]. The mechanism of action of HSP70 chaperones is based on their ability to cycle through conformational changes in an ATP-dependent manner under the regulation of the chaperones of the HSP40 (DnaJ) family and nucleotide exchange factors (NEF). Briefly, HSP40 chaperones recognize and deliver non-native proteins that expose hydrophobic regions to HSP70s, temporarily blocking their aggregation propensity. When ATP is hydrolyzed to ADP, the peptide is stably bound. The return of the ATP-bound state will allow the release of the substrate [30,31,32,33,34].

A member of the HSP70 family, well-conserved among eukaryotes, is BiP, which, along with lectin chaperones, is the master of ER quality control (ERQC). BiP recognizes nascent polypeptide chains of both glycosylated and non-glycosylated proteins and can bind to unfolded substrates in an ATP-dependent manner. This process of binding and release is assisted by co-factors such as the ER-localized DnaJ-like proteins (ERdjs) and two NEF named GRP170 and Sil1 [35,36].

HSP40 proteins, also known as JDPs (J domain proteins), are encoded by 50 genes in the human genome [27]. They are all characterized by the presence of a ~70 amino acid J domain highly conserved and have been divided into three classes, A, B and C (previously named class I, II and III). JDPs from class A and B also share a glycine-phenilalanine-rich region (G/F) and two sandwich domains at the C-terminal, while only class A present a Zn-binding domain. The class C proteins have little homology with one another, only sharing the J domain, which could be located at any place in the protein sequence [32,33,34].

HSP90/HSC family proteins are encoded by six genes in the human genomes and function as a homodimer. Each HSP90 monomer is composed of three highly conserved domains, an N-terminal domain (NTD) implicated in the binding of ATP, a middle domain (MD) necessary for the ATP hydrolysis and a C-terminal domain (CTD) with a MEEVD motif for co-chaperone coupling. Upon binding and hydrolysis of ATP, HSP90 cycle through different conformational states with different affinity for HSP70, the adaptor protein Hop/Sti1 and the protein client to fold [37,38]. About 10% of human proteins are HSP90 clients, depending on them for their maturation [30].

Chaperones of the HSP110 family are encoded in the human genome by four genes and are highly homologous to the HSP70 family. Three of these proteins (HSPH1-3 according to a revised nomenclature [27]) are both nuclear and cytosolic, while the fourth HSPH4, also known as GRP170 or HYOU1, is located in the SR/ER where it covers a fundamental role in the ERQC, also acting as NEF for BiP [30,39].

To date, 11 small HSPs chaperones have been found located in the cytosol and nucleus of mammalian cells and are highly upregulated under cellular stress. These small HSPs are characterized by a conserved crystallin domain, with N- and C-terminus that can vary and might oligomerize with different members of the family, thus increasing their chaperone specificity [27,40].

Additionally, two types of HSP60 proteins, also known as chaperonins, have been found in different subcellular locations. Group I members (HSP60 in eukaryotes, GroEL in prokaryotes) have a double-layered, 7-subunit ring configuration and are located in the eukaryotic mitochondria or chloroplasts. They can bind cofactor HSP10 (GroES in prokaryotes) in an ATP-dependent manner. Group II members are composed of double-layered, 8,9-subunit rings and are located in the cytosol (also known as TRiC or CCT). These large double-ring complexes can encapsulate their client molecule one at a time in the central cavity for folding, lowering the risk of aggregation [30,41,42].

### 2.2. Alternative PQC: Autophagy and Aggrephagy

If the misfolded state of proteins becomes irreversible, then the ubiquitin-proteasome and the autophagy systems intervene for their degradation.

The initial step for both mechanisms is the ubiquitination of the cargoes that have to be recognized and targeted for degradation. Ubiquitin is a small regulatory protein (76 amino acids) [43] that was first described in 1975 by Goldstein and colleagues [44]. It is part of a family of proteins with diverse amino acid sequences but sharing similar folding structures [45,46]. Ubiquitin is found in all eukaryotes but not in prokaryotes, and its sequence is remarkably conserved between humans and yeast, with a difference of only three amino acids.

The cargoes’ ubiquitination occurs at lysine residues via an isopeptide bound with the C-terminal Gly of ubiquitin. Furthermore, ubiquitin itself has seven lysine residues (K6, K11, K27, K29, K33, K48 and K63) and an N-terminal methionine (M1) which in turn can be ubiquitinated. Moreover, ubiquitin can undergo ubiquitin-like modifications such as SUMOylation and NEDDylation, thus generating a wide range of signaling codes. Conjugation of ubiquitin occurs in a three-step cascade sequentially involving three classes of enzymes: the ubiquitin-activating enzyme E1, the ubiquitin-conjugating enzyme E2 and a substrate-specific ubiquitin-protein ligase E3. Another group of enzymes called deubiquitinating enzymes (DUBs) is responsible for the removal of ubiquitins from their substrates.

For a more complete overview of ubiquitin, its structure and modifications, we suggest some comprehensive reviews to look at in citations [46,47,48,49,50,51,52,53,54,55,56].

Notably, ubiquitination at Lys11 and Lys48 residues is usually a signal for the elimination of the targeted cargoes through the proteasome, a large multicomplex of 2.5 MDa composed of the 20S catalytic core particle (CP), and one or two 19S regulatory particles (RPs). For an in-depth description and characterization of the 26S proteasome, we refer to some key papers [47,51,57,58,59,60,61,62].

Before proteasome degradation could proceed, misfolded proteins should be unfolded and fit through the narrow pore of the 20S subunit. If this cannot occur, ubiquitinated proteins are targeted for autophagy degradation. In particular, the ubiquitination of Lys68 seems to be preferentially a signal for degradation through this pathway [63].

It is nowadays clear that UPS and autophagy are interconnected, sharing some of their components, and the perturbation of one system affects the other. For instance, proteasome inhibition induces activation of autophagy as a compensatory system in cell lines [64,65] as well as in in vivo experiments in genetically modified *Drosophila melanogaster* [66].

The term autophagy was first conceived by biochemist Christian de Duve, a Nobel Prize laureate in Physiology or Medicine of 1974, for his discovery of lysosome. In 2016, another Nobel Prize in Physiology or Medicine was awarded to the cell biologist Yoshinori Ohsumi for his in-depth studies on the autophagy machinery and the discovery of Autophagy-related genes (Atg) [67]. In mammalian cells, three types of autophagy are characterized, chaperone-mediated autophagy (CMA), microautophagy and macroautophagy. We will particularly focus on macroautophagy (henceforth autophagy) and its specialized form of selective removal and degradation of protein aggregates termed aggrephagy by Seglen and coworkers in 2007 [68].

Autophagy is a tightly regulated process necessary for the maintenance of proper cellular homeostasis by bulk degradation of cytoplasmic content during nutrient starvation or the removal of specific elements such as damaged organelles, lipids, pathogens and, more importantly, protein aggregates.

Activation of autophagy involves the formation of an isolation membrane, the phagophore, which expands into a double-membrane vesicle, the autophagosome, to engulf what needs to be recycled. The fusion with a lysosome generates an autolysosome in which the lytic enzymes can carry out degradation [69]. In brief, initiation of autophagy is achieved by the ULK complex comprised of, in mammals, ULK1or ULK2 as well as the mammalian homologs ATG13, ATG101 and RB1 inducible coiled-coil 1 (RB1CC1, also known as FIP200). The following nucleation step is controlled by the ATG14-containing class III phosphatidylinositol 3-kinase (PtdIns3K) complex, consisting of PIK3C3/VPS34, PIK3R4/VPS15, BECN1, the nuclear receptor binding factor 2 (NRBF2) and the membrane curvature sensor ATG14. The phagophore expansion step then can proceed with regulation from the ATG12-ATG5-ATG16L1 complex and the Atg8/LC3 complex composed of different subfamilies and their isoforms (LC3 family including LC3A, LC3B, LC3B2 and LC3C and GABARAP family including GABARAP, GABARAPL1 and GABARAPL2). Closure of the membrane and fusion with a lysosome will form a mature autolysosome [67,70,71,72,73].

Molecular regulation of autophagy involves a complex yet beautifully harmonized cascade of events that is initiated by inhibition of the mechanistic target or rapamycin mTOR, which is composed of two complexes, mTOR complex1 and mTOR complex2 (mTORC1 and mTORC2). Likewise, the energy-sensing AMP-activated protein kinase (AMPK) is also involved in the initiation of autophagy by the inhibition of mTOR and activation of ULK1 through its phosphorylation. Many detailed studies illustrate in depth these mechanisms [70,74,75,76,77,78,79,80,81].

Notably, autophagy in skeletal muscle is regulated by transcription factors such as Runx1 and the members of the Forkhead Box O family (FoxO1, 3 and 4), which control the expression of autophagy genes like LC3 and VPS34, among others. FoxO transcription factors are, in turn, downstream targets of the kinase Akt [11,82,83]. Very recently, a new FoxO-dependent regulator of autophagy in skeletal muscle has been identified by Leduc-Gaudet and colleagues. This regulator, named MYTHO (Macroautophagy and YouTH Optimizer) by the authors, was found to co-localize with LC3 and, in part, with LAMP2. Moreover, with knockdown experiments, MYTHO proved to be essential for autophagy, leading, upon long-term silencing, to severe myopathic features with inflammation, degeneration/regeneration, mitochondria swelling and tubular aggregates. Furthermore, the authors showed that MYTHO’s expression is also downregulated in skeletal muscle biopsies from dystrophy type 1 (DM1) patients, which are characterized by similar myopathic histopathological features [84].

It is well understood that autophagy is not solely a non-selective invagination of cytoplasmic portions induced upon starvation but also could be a highly specialized mechanism able to remove many different selected elements within the cell, relying on cargo receptors. These processes have been named based on the element that is targeted for degradation, and they include, among others, the elimination of damaged or old mitochondria (mitophagy), nuclei (nucleophagy), ribosomes (ribophagy), lipids (lipophagy), lysosomes (lysophagy) and also protein aggregates (aggrephagy) [85,86,87,88,89,90].

When misfolding becomes irreversible, proteins might form oligomers and interact with other unfolded proteins, thus producing larger insoluble protein aggregates within the cytoplasm. These aggregates can organize into histologically diverse structures such as spheres, tangles or threads, either amorphous or structured, similar to amyloids [91,92]. These aggregates, named aggresomes by Kopito and colleagues [93], have been found to localize in a region near the nuclei, the microtubule-organizing center (MTOC), surrounded by a structure resembling a cage constituted by the intermediate filament vimentin [92,93,94]. Protein aggregates are delivered to the aggresome by the motor protein dynein that, facilitated by the mediation of the histone deacetylase 6 (HDAC6), moves the polyubiquitinated proteins along the microtubules [95,96].

### 2.3. Key Proteins in Aggrephagy

**Figure 1:**
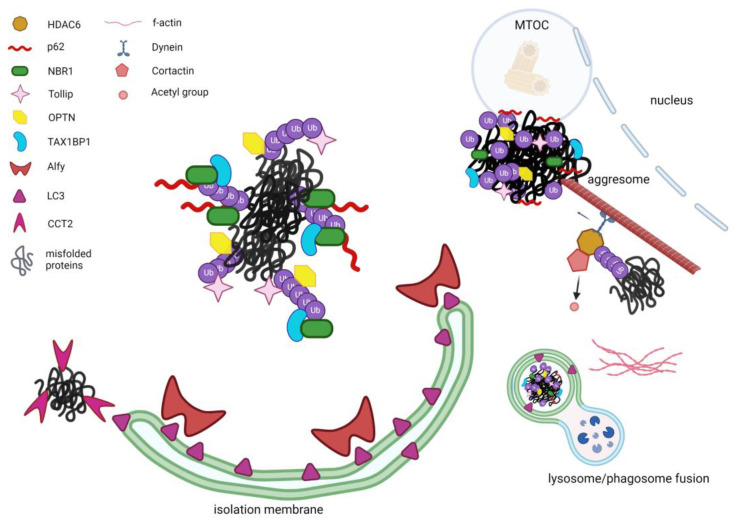
Schematic representation of the known aggrephagy modulators. Misfolded ubiquitinated proteins are recognized by the selective aggrephagy receptors such as p62 and NBR1 which jointly associate through their UBA domains to the ubiquitins that tag misfolded proteins. Moreover, via their LIR domains they interact with LC3. Tollip, optineurin and TAX1BP1 can also recognize ubiquitinated aggregates, with TAX1BP1 being mobilized to the aggregates by NBR1. The large protein Alfy can act as a scaffold, stabilizing the forming phagophore and interacting with LC3 and p62. HDAC6 is also facilitating the selective aggregates’ removal by bridging the ubiquitinated proteins to the microtubule motor dynein, which can guide the aggregates toward the microtubule organization centre (MTOC) where aggresomes are localized. Furthermore, HDAC6 recruits and de-acetylates cortactin which, once activated, can assemble an F-actin network surrounding aggregates and lysosome, thus facilitating the fusion between lysosome and utophagosome. Likewise, CCT2 in its monomeric form can recognize aggregated proteins independent of ubiquitin-binding receptors and direct them towards the nascent autophagosome through interaction with LC3 via its VLIR motif. (Figure created with Biorender.com, Toronto, ON, Canada).

Since the first descriptions of the aggresomes and aggrephagy pathway as a specialized form of autophagy, the interest of many researchers has focused on the identification of the specific players that could possibly orchestrate this fine mechanism (Figure 1). Structures, functions and interactors described in this paragraph are summarized in Table 1.

#### 2.3.1. HDAC6

One of the first studied proteins involved in the removal of aggregates is the cytoplasmic histone deacetylase 6 (HDAC6), a microtubule-associate deacetylase [97] containing a ubiquitin-binding domain (BUZ) and two catalytic domains (DD1 and DD2). Kawaguchi and colleagues [95] showed that, in an in vitro system, HDAC6 interacts with polyubiquitin-positive aggresomes via its BUZ domain. It can also associate with the motor protein dynein through its dynein motor binding domain (DMB). Moreover, it was demonstrated that HDAC6 mediates the recruitment of lysosomes to the MTOC, thus allowing the removal of aggregated Huntingtin in vitro [98]. It was also discovered that, in *Drosophila melanogaster*, HDAC6 is crucial for the compensatory role of autophagy when the UPS system is impaired [66]. HDAC6 also mediates the aggresomes removal, facilitating autophagosomes and lysosomes fusion by activating cortactin (through its deacetylation), an actin-remodeling factor that stimulates the local assembly of an F-actin network [99]. HDAC6 has been found to accumulate in Lewy bodies of brain sections of Parkinson’s disease (PD) patients, along with α-synuclein and ubiquitin [95].

#### 2.3.2. P62/SQSTM1

Sequestosome 1, also known as p62, is the first selective autophagy receptor that was described to be capable of binding polyubiquitinated proteins [100] as well as binding directly to LC3 and other human homologues of Atg8 [101]. Mutations in the p62 gene have been linked to diverse human diseases such as amyotrophic lateral sclerosis (ALS), frontotemporal dementia (FD), neurodegeneration with ataxia and also distal myopathy with rimmed vacuoles and Paget disease of the bone [102,103,104,105,106]. P62 is ubiquitously expressed and is able to shuttle between the nucleus and cytoplasm [107] and is characterized by specific domains that shape its functions. It contains a Phox1 and Bem1p (PB1) domain necessary for homo or hetero-oligomerization, thus mediating the formation of protein aggregates, a ZZ-type zinc finger (ZZ) domain, two nuclear localization signal (NLS) sequences and a nuclear export signal (NES) sequence, an LC3-interacting region (LIR) and a C-terminal ubiquitin-associated (UBA) domain. Homodimerization via the UBA domain keeps p62 inactive [108], while phosphorylation of the Ser407 is able to reverse it to an active monomeric form [109]. Thus, through its PB1 domain, p62 is able to bind ubiquitinated proteins, forming long helical filaments [110] that are next targeted for autophagy degradation via the binding to LC3 with its LIR domain [101,111]. Two independent studies [112,113] have demonstrated that in in vitro experiments, p62 is able to phase separate ubiquitinated proteins and that the p62-decorated aggregates have liquid-like features and are able to exchange their components.

#### 2.3.3. NBR1 (Neighbor of BRCA1 Gene 1)

NBR1 is a soluble selective autophagy receptor that was discovered soon after p62 for its ability to bind to LC3 and the presence of a similar structural organization [114,115]. It is evolutionarily conserved and precedes the existence of p62 since most non-metazoans only contain NBR1 but not p62 [116]. Its structure contains a PB1 domain which is able to bind to p62, a ZZ domain, an LIR motif for the interaction with LC3 and a C-terminal UBA domain [114,117]. Moreover, unlike p62, it contains four tryptophan (FW) domains allowing its binding to microtubule-associated protein MAP1B and TAX1BP1 [118], as well as an amphipathic helix (HA) domain that, together with the UBA domain, is necessary for co-localization with LAMP2 [119]. During the removal of aggregates, NBR1 uses its PB1 domain to bind p62 and its UBA domain to enhance the binding to ubiquitinated cargoes. Meanwhile, TAX1BP1 is recruited through its FW domain [113,120]. In the muscle, NBR1 directly interacts with the serine/threonine kinase domain (TK) of the protein titin, a domain that regulates muscle gene expression. Two mutations have been identified in this domain, causing disruption of the interaction between NBR1 and titin and leading to core myopathy, probably the most common form of congenital myopathies in childhood [121,122,123,124].

#### 2.3.4. WDFY3 (Alfy)

The link between Alfy (autophagy-linked FYVE protein) and protein aggregates’ removal was first demonstrated in 2004 [125]. Alfy is a 400 kDa protein, evolutionarily conserved and expressed in all tissues. It contains five WD40 repeats and a PH-BEACH domain assemblage, and a C terminal phosphatidylinositol-3-phosphate (PI3P)-binding FYVE domain; therefore, it is also known as WDFY3. Alfy was found to co-localize with the p62-positive aggregates, and, in vitro, this interaction was found to be mediated by the region comprising amino acids 170–206. It is required for aggrephagy but not for starvation-induced bulk autophagy [90,126,127,128]. Due to its large size, it is considered a scaffold protein facilitating the assembly of p62 bodies and their incorporation in autophagosomes. It was demonstrated that overexpression of Alfy decreases the level of polyglutamine aggregates in a primary neuronal HD model and in an in vivo model of eye polyglutamine disease in *Drosophila* [126], thus highlighting its potential therapeutic value.

#### 2.3.5. TOLLIP (TOLL-Interacting Protein)

Tollip is the mammalian homolog of the yeast Cue5. It is a member of the CUET family of proteins and therefore is characterized by a ubiquitin-binding CUE domain and an LC3-interacting region (LIR). It was demonstrated that Cue5 and Tollip are able to promote the clearance of the aggregate of human huntingtin mutant and that Tollip is more efficient than p62 in the clearance of aggregates in HeLa cells [129].

#### 2.3.6. Optineurin

Optineurin is a cytosolic protein ubiquitously expressed and with numerous cellular functions such as vesicle trafficking, cell division control, and autophagy. It is linked to numerous diseases, like amyotrophic lateral sclerosis and primary open angle glaucoma (POAG) [130,131,132,133,134]. Optineurin is constituted by a NEMO-like domain, an LC3-interacting (LIR) domain, an LZ domain, a ubiquitin-binding domain (UBD), coil-coiled (CC) domains, and a zinc-finger-like (ZnF) domain. It was first demonstrated [135] that lack of optineurin in HeLa cells increases aggregation of SOD1 G93C and huntingtin mutant, revealing its involvement in the aggregates’ clearance. Moreover, Shen and colleagues demonstrated that [136], in transgenic mice, the co-localization of optineurin with huntingtin mutant inclusion bodies requires its UBD domain. The overexpression of optineurin caused a reduction in inclusion bodies through polyubiquitin-mediated autophagy, while mutation of the UBD domain caused inclusion body accumulation.

#### 2.3.7. TAX1BP1 (Tax1-Binding Protein 1)

TAX1BP1 involvement in the clearance of aggregates was first discovered in 2012 by Newman and colleagues [137], and it was more recently demonstrated that loss of TAX1BP1 induces the formation of proteotoxic aggregates in the brain [138]. TAX1BP1 has an N-terminal SKIP carboxyl homology (SKICH) domain, a central oligomerization domain containing three coiled-coil (CC) regions, and a C-terminal ubiquitin binding domain (UBD) containing two zinc fingers [139]. Its direct interaction with NBR1 allows its recruitment to the aggregates and, in turn, TAX1BP1 is able to recruit the autophagy factor FIP200 (an ULK-interacting protein also known as RB1-inducible coiled-coil protein 1, RBCC1) via the Claw domain, allowing autophagosome formation [120].

#### 2.3.8. CCT2 (Chaperonin Containing TCP1 Subunit 2)

Very recently, Ma and colleagues [140] identified a new function of the chaperonin subunit CCT2 in the elimination of aggregates. The chaperonin TCP-1 ring complex (TRiC) is constituted by eight subunits (CCT1-8) and each one contains an equatorial domain responsible for the binding of ATP as well as an apical domain responsible for substrate binding. In this work, the authors highlighted a newly discovered function of the CCT2 subunit as an aggrephagy receptor, facilitating protein aggregates’ engulfment into the autophagosome and their subsequent clearance. This function is independent from other aggrephagy proteins such as p62, NBR1 and TAX1BP1 and is mediated by the interaction with the ATG8 proteins. The mechanism of action and the ability to switch between functions, from protein-folding to autophagy, is conveyed by its monomeric form, which exposes the ATG8-interaction motif. The authors speculate that under physiological conditions, CCT2 work as a subunit of TRiC to carry out protein folding function. However, in the presence of excessive aggregation-prone proteins, CCT2 becomes monomeric to promote aggregate removal through autophagy. CCT2 does not bind poly-ubiquitinated proteins.

**Table 1:** Summary of the aggrephagy key players and their different cellular functions.

Protein	Function/Structure	Main Functions in Aggrephagy	Interactors
**HDAC6**	Microtubule-associate deacetylase. BUZ domain; DD1 and DD2 catalytic domains.	- Mediates the recruitment of lysosomes to the MTOC - Mediates the aggresomes removal, facilitating autophagosomes and lysosomes fusion by activating cortactin (through its deacetylation)	- Polyubiquitin-positive aggresomes - Dynein
**P62/SQSTM1**	Receptor for polyubiquitinated proteins. PB1 and ZZ domains; Two NLS and one NES sequences; LIR motif; C-terminal UBA domain.	- Mediates the formation of protein aggregates - Bind ubiquitinated proteins, targeted for autophagy degradation	- Polyubiquitinated proteins - LC3
**NBR1**	Soluble selective autophagy receptor. PB1, ZZ and C-terminal UBA domains; LIR motif; FW and HA domains.	- Implied in the removal of aggregates - Bind microtubule-associated protein MAP1B and TAX1BP1 - Co-localization with LAMP2	- LC3 - p62 - Polyubiquitinated proteins - Microtubule-associated protein MAP1B and TAX1BP1 - Titin
**WDFY3 (ALFY)**	Scaffold protein, evolutionarily conserved and expressed in all tissues. Five WD40 repeats PH-BEACH domain assemblage; C terminal PI3P-binding FYVE domain.	- Facilitates the assembly of p62 bodies and their incorporation in autophagosomes	- p62-positive aggregates
**TOLLIP**	Member of the CUET family of protein. Ubiquitin-binding CUE domain; LIR motif.	- Crucial for removal of protein aggregates	- LC3 - NBR1 - Polyubiquitinated proteins
**OPTINEURIN**	Cytosolic protein ubiquitously expressed implies in cellular functions such as vesicle trafficking, cell division control, and autophagy. NEMO-like, LZ, UBD, CC and ZnF domains; LIR motif.	- Involves in the aggregates’ clearance - Reduces inclusion bodies through polyubiquitin-mediated autophagy	- LC3 - Inclusion bodies
**TAX1BP1**	Protein implies in regulation of cell death and inflammatory signalling pathways. N-terminal SKICH domain; Central oligomerization domain containing three CC regions; C-terminal UBD containing two zinc fingers.	- Involves in clearance of aggregates - Facilitates autophagosome formation	- NBR1 - Ubiquitinated protein aggregates
**CCT2**	Chaperonin subunit Implies in protein-folding and autophagy ATG8-interaction motif	- Aggrephagy receptor, function conducts in the monomeric form - Facilitates protein aggregates’ engulfment into the autophagosome and their subsequent clearance	- LC3

BUZ: Ubiquitin-Binding BUZ, PB1: Phox1 And Bem1p, ZZ: Zz-Type Zinc Finger, NLS: Nuclear Localization Signal, NES: Nuclear Export Signal, LIR: Lc3-Interacting Region, UBA: Ubiquitin-Associated, FW: Four Tryptophan, HA: Amphipatic Helix, PI3P: Phosphati-Dylinositol-3-Phosphate, UBD: Ubiquitin-Binding, Cc: Coil-Coiled, ZnF: Zinc-Finger-Like, SKICH: Skip Carboxyl Homology.

### 2.4. Aggrephagy Players as Therapeutic Targets

Defective autophagy is known to be linked to various human diseases, such as neurodegenerative, cardiovascular, metabolic, autoimmune diseases and cancer, as well as aging [141,142,143,144,145,146]. Various drugs targeting macroautophagy exist, and although their pharmacological effects are non-specific, they have been approved in the clinical practice for diseases other than myopathies. Their mechanism of action and use in clinical settings or in preclinical studies have been recently reviewed [147,148]. Among the activators of autophagy, rapamycin (known as sirolimus) and its analogs (rapalogs), such as everolimus, tacrolimus and temsirolimus, to name some, act by allosterically inhibiting the kinase activity of mTORC1. These compounds have already been approved to prevent transplant rejection [149], for the treatment of some types of cancers (like pancreatic neuroendocrine tumors and renal cell carcinoma) [150] and moreover, a phase II randomized multicenter clinical trial is undergoing to test the efficacy of the use of rapamycin in combination with riluzole for the treatment of amyotrophic lateral sclerosis (NCT03359538). Other compounds, Torin1 and 2, found to activate autophagy by targeting the ATP binding site of both mTORC1 and 2, are promising drugs to be used in the clinic [151,152]. Similarly, inhibitory drugs have also been developed. Among these are chloroquine and hydroxychloroquine, two lysosomotropic agents that, in combination with anticancer drugs, radiotherapy or surgery, are used for the treatment of solid and hematological tumors [153].

Despite many compounds being known to modulate autophagy and, therefore, could be potential drugs for those diseases in which autophagy is impaired, more studies are needed to fully clarify their exact mechanism of action and, moreover, to reduce the tissue’s toxicity, especially in chronic administration.

To date, no treatments specifically targeting the aggrephagy exist. Therefore, the development of such selective drugs that might increase efficacy and/or reduce side effects represents a fundamental and undeniable urgency.

However, pharmacological discovery in this field shows some challenges, including the lack of aggrephagy-specific biomarkers and the need for appropriate in vivo models.

Thus far, only a few molecules have been designed and tested in vitro to inhibit autophagy for the treatment of various types of cancers. Specifically, p62 has been identified as a possible target, and two different molecules have been developed. Kalid and colleagues [154] showed that PTX80, a compound that was retrospectively identified as a ligand to p62, causes proteotoxic stress-inducing cell apoptosis by reducing the solubility of p62 and, as a consequence, its ability to co-localize with ubiquitinated proteins which will therefore accumulate in the cytoplasm. In another work by Lee and colleagues [155], a small molecule named YOK-1104, able to bind to the ZZ domain of p62, was shown to be a promising drug to be used in combination with irradiation for the induction of apoptosis in those cancer cells that are resistant to cell death.

## 3. Aggrephagy Involvement in Muscle Diseases

Aggrephagy involvement has been studied and described in a few individual cases or a small case series of genetic and sporadic muscle diseases. Clinical phenotypes, age of onset, disease course and systemic involvement reported in these cases are heterogeneous and summarized in Table 2.

### 3.1. Myotilinopathies and Desminopathies

**Figure 2:**
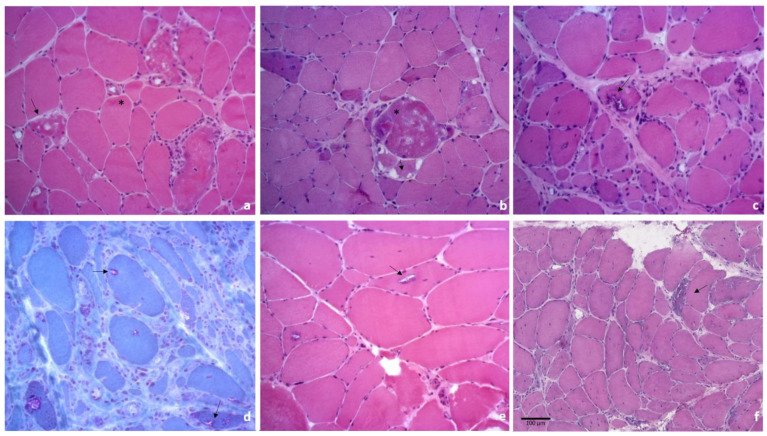
Histology from muscle biopsies of patients affected by muscle diseases with aggrephagy involvement. (**a**) hematoxylin/eosin (H&E) staining of a muscle biopsy from a patient affected by a mutation in the myotilin gene highlighting fiber size variability, intracitoplasmatic and subsarcolemmal eosinophilic areas (*), vacuoles, either rimmed or not (arrows); (**b**) H&E of a muscle biopsy from a patient with a mutation in the desmin gene showing fiber size variability, with concomitant atrophy and hypertrophy, some fibers present basophilic and granular cytoplasmic material (*) and vacuoles (arrows); (**c**,**d**) histological findings in a muscle biopsy of an sIBM patient, showing atrophic and hypertrophic muscle fibers, with scattered and groups of small angulated fibers, fiber with rimmed vacuoles and vacuoles with a rim of granular basophilia in H&E (**c**), that is stained in red with Gomori trichrome (**d**); (**e**) muscle biopsy from a patient affected by OPMD, demonstrating variability in fibers size, rare internal nuclei, scattered atrophic and hypotrophic fibers and rimmed vacuoles (arrows); (**f**) muscle biopsy from a patient affected by *PLIN4*-related myopathy demonstrating numerous degenerated fibers, frequent central nuclei, fibers splitting and vacuoles (arrow).

Myofibrillar myopathies (MFMs) represent a broad hereditary group of muscle disorders characterized by myofibrillar alterations, Z-disk disorganization, sarcoplasmic buildup of myofibrillar degradation products, often with the presence of rimmed vacuoles, and accumulation of multiple proteins in the muscle tissue (Figure 2a,b). Therefore, MFMs diagnosis is based on histological features since the clinical spectrum is relatively wide.

Genetically, the cause of MFMs is highly heterogeneous and associated with genes encoding for Z-disk-related proteins, such as desmin, aB-crystallin, myotilin, ZASP, filamin c and BAG3 [156,157,158].

Recently, due to the use of next-generation sequencing, patients with MFM phenotypes have been found to carry mutations in new genes, such as *SVIL* [159] and *UNC45B* [160]. Additionally, known genes related to different disorders, such as *FHL1*, *DNAJB6*, *VCP*, *HSBP8*, *TTN*, *ACTA1*, *LMNA*, *PLEC*, *KY*, *PYROXD1* and p62 + *TIA1*, have now been associated with MFM phenotypes [5,105,161,162,163,164].

Protein aggregates in MFMs have been found to contain a great number of different proteins, including phosphorylated tau (p-tau) and β-amyloid, and have also been associated with increased immunoreactivity for the proteasome 19S and 20S subunits [165,166]. Prompted by these observations, Olivé and colleagues [167] examined and first reported in 2008 the presence of p62 and UBB + 1, a mutant form of ubiquitin B known to accumulate in neurodegenerative disorders such as Alzheimer’s and Huntington’s disease, in a cohort of myotilinopathy and desminopathy patients. In their analyzed samples, twelve myotilin and five desmin-mutated patients, the muscle biopsies showed the presence of protein aggregates within the cytoplasm or in the subsarcolemmal region of the muscle fibers as revealed by the modified Gomori trichrome staining. All the myotilinopathies muscles also displayed a large number of vacuoles as well as spheroid bodies, strongly positive for Thioflavin T staining, while in the desminopathies muscles, only a few cytoplasmic bodies and vacuoles were visible, and no Thioflavin T positivity was present. In this study, all biopsy samples showed immunoreactivity for p62, with stronger signals present in myotilinopathy samples than in desminopathy samples. UBB + 1 was also found to be activated even though to a lesser extent and with a higher variability between patients. Similar to p62, fainter signals were found in desminopathy samples compared to myotilinopathy ones. These findings led the authors to conclude that the pathogenic mechanism in these MFM might indeed be due to an impairment of the UPS, while p62 upregulation could be interpreted as a compensatory response to the possibly toxic protein aggregates.

### 3.2. Sporadic Inclusion Body Myositis

sIBM is the most recurrent sporadic muscle disease in older populations, with the slow progression of muscle impairment accompanied by muscular atrophy. It is still categorized as idiopathic inflammatory myopathies, similar to polymyositis and dermatomyositis, which are characterized by endomysial inflammation associated with various degenerative changes of the muscle fibers (Figure 2c,d) and heterogeneous muscle impairment [168]. Although the lack of response to immunosuppressive or immunomodulatory therapies clearly suggests that the disease is mainly degenerative, and the inflammatory component role is usually limited in early stages [169,170].

sIBM generally shows CD8+ T cells invasion of non-necrotic muscle fibers, incremented expression of MHC class I, rimmed vacuoles, and protein aggregates which could contain, among others, beta amyloid protein, p62 and TDP-43, like those found in Alzheimer’s disease, in amyotrophic lateral sclerosis and frontotemporal dementia [171,172].

Prof. Valerie Askanas’s group investigated the expression of proteins involved in the selective removal of aggregates in muscle biopsies of sIBM patients defined by the typical clinical features [173,174]. Their interest arose from the observation of a certain degree of phenotypic similarity between the sIBM muscle tissue and the brain of Alzheimer’s and Parkinson’s disease patients. The group showed that in sIBM but not in control samples (including polymyositis, dermatomyositis, amyotrophic lateral sclerosis, peripheral neuropathy patients and normal controls), there is increased positivity for p62, LC3 and NBR1. Specifically, in 80% of the vacuolated and 20–25% of the non-vacuolated fibers, p62 strongly accumulates within the aggregates, co-localizing with ubiquitin and p-tau [175]. Moreover, p62, even though in close proximity to, did not appear to co-localize with LC3, which was also found to be activated and accumulating in the cytoplasm of sIBM biopsies [176]. In a later work, the involvement of NBR1 was also considered [177], demonstrating that this aggrephagy adaptor is likewise present within the muscle fibers, accumulating in the form of aggregates and co-localizing with p62, LC3, ubiquitin and p-tau. Furthermore, Nicot and colleagues [178] identified, by in vitro studies, a posttranslational modification of NBR1 by the glycogen synthase kinase 3 (GSK3), which will regulate its activity and showed that a strong dephosphorilation of NBR1 is present in the muscle tissues of sIBM patients, which directly correlates with the severity of the phenotype.

Two different theories exist for the primary mechanism involved in the pathogenesis of sIBM, an autoimmune activation causing T cell-mediated cytotoxicity with consequent damage of the muscle fibers or a degenerative pathway activated by defective aggrephagy. The non-responsiveness to the immunosuppressive therapies and the observations described above seem to better support the initial involvement of a defective protein degradation in the pathogenesis of sIBM [179].

### 3.3. Rigid Spine Syndrome with FHL1 Mutation

Reducing body myopathy due to *FHL1* mutations is an X-linked disorder, marked by the presence of intracytoplasmic inclusion bodies that contain a multitude of proteins, among which mutant FHL1 protein is the most represented.

In patients affected by rigid spine syndrome (RSS) with a mutation in the *FHL1* gene, Bonaldo’s group described for the first time the activation of the pathway involved in the selective removal of aggresomes [180]. In about 20% of the muscle fibers of the proband and her brother’s biopsies, the authors identified reducing bodies (RBs) that were Congo-red positive, thus indicating an amyloid-like nature and also revealed positivity for the FHL1 antibody. Further analysis of these aggregates showed co-localization between FHL1 and p62, as well as the presence of DAPI-positive material and ubiquitin within the aggregates. Positivity for LC3 was also detected, although no co-localization with the FHL1 signal was observed. Doing Western blot on muscle protein extract, Beclin1 and BNIP3, two autophagy enhancers, were proven to be strongly induced. A slight increase in the lipidated form of LC3 (LC3-II) was also detected. The identified mutation, a cysteine to arginine conversion in position 150, fell in the second LIM domain of FHL1, which is predicted to promote misfolding of the protein. Previous reports [181,182] of patients with the same p.C150R mutation also show the presence of RBs positive for FHL1 staining. Therefore, the authors conclude that all the data presented strongly indicate the involvement of the aggresome and the autophagy machinery in the muscular pathological mechanism underlying RSS patients with p.C150R *FHL1* mutation.

### 3.4. Oculopharyngeal Muscular Dystrophy

Oculopharingeal muscular dystrophy has mainly autosomal dominant inheritance with progressive late onset of proximal limb, facial and extraocular muscle weakness due to a poly-alanine expansion in the Poly(A) Binding Protein Nuclear 1 (PABPN1) [183,184].

It has been demonstrated that PABPN1 plays a critical role in mRNA biogenesis and myogenesis. In fact, PAPBN1 is implicated in post-transcriptional RNA processing, including long non-coding RNAs (lncRNAs) [185]. Despite being ubiquitous, its alteration results in a muscle-specific disease, with defective myoblast proliferation and differentiation correlated with shorter mRNA poly(A) tails and subsequent nuclear accumulation of poly(A) RNA and misfolded PABPN1.

Thus, it has been postulated that the pathogenetic mechanism involves a reduced bioavailability of PABPN1 due to its aggregation [184].

OPMD muscle biopsies show fiber size variability, rare internal nuclei, scattered atrophic and hypotrophic fibers and rimmed vacuoles (Figure 2e).

In a cohort of 21 Chinese patients clinically affected by OPMD and genetically characterized by five different pathological genotypes, Lin and colleagues investigated the involvement of the autophagy pathway, focusing on the physical association between PABPN1 and p62, NBR1, LC3 and the ubiquitinated protein marker FK2 [186]. In two patients’ muscle biopsies, the authors identified abnormal accumulation of PABPN1 present in 5–7% of the muscle fibers, which were also stained positive for p62, NBR1 and FK2 and, to a lesser extent, LC3. OPMD nuclear inclusions have been reported to contain ubiquitin and the chaperone HSP70 [187,188]. Therefore, the authors conclude that a pathogenic mechanism related to protein degradation involving both the UPS and the selective removal of aggregates is underlying OPMD.

### 3.5. PLIN4-Related Myopathy

Recently, in an Italian family affected by a late onset myopathy, with distal or less frequently limb-girdle muscle involvement and autophagic features within the muscle, we identified by multi-omic approach a potentially causative large coding expansion in the *PLIN4* gene encoding for perilipin 4 [8]. Perilipin 4 is part of a family of proteins involved in the metabolism of lipids, protecting them from lipases by coating the lipid droplets (LDs). Its structure is more divergent compared to the other perilipins, containing a large amphipathic helix responsible for the interaction with the lipids, which is organized in a 3–11 alpha helix structurally similar to α-synuclein and apolipoproteins. The identified genetic mutation causes a 99 nt coding region to be repeated nine more times compared to the wild-type, thus adding 297 extra amino acids to the encoded protein.

Numerous degenerated fibers, frequent central nuclei, fibers splitting and vacuoles (Figure 2f) are observed in patients’ muscle biopsies.

The pathogenic mechanism involved is indicated to be the aggregation of the mutated protein with consequent activation of the aggrephagy pathway for the aggregates’ removal, as demonstrated by the co-localization between perilipin 4 signal and FK2, p62 and NBR1 as well as by the increased signal of WDFY3. The aggregates are present at the subsarcolemmal region of the muscle fibers and within the vacuoles, and positivity for these antibodies is directly correlated with the severity of the phenotype, thus indicating a progressive blockage and inefficiency of the aggrephagy mechanism. More recently, three unrelated families and one Chinese sporadic patient with *PLIN4*-related myopathy have been reported [189,190]. In these reports, the same activation of the aggrephagy pathway has been confirmed by immunostaining of the muscle biopsies. Notably, one family with a more severe and more rapidly progressing proximal phenotype was associated with a longer repeat expansion [189].

**Table 2:** Clinical, laboratory and histological characteristics of muscle disorders with reported aggrephagy markers.

Disease	Gene/ Inheritance	Age at Onset	Predominant Muscle Involvement	Systemic Involvement	Evolution, Loss of Ambulation	Creatin Kinase Level	Muscle Biopsy: Rimmed Vacuoles and Protein Aggregates
Myofibrillar myopathy [156,157,158,167]	*MYOT*/AD *DES*/AD	Adult Young adult	Distal Distal + limb-girdle	Rarely cardiomyopathy and peripheral neuropathy Cardiomyopathy	Slowly progressive Slowly progressive, LoA in later stages	Mild to highly elevated	Rimmed vacuoles and protein aggregates +
Inclusion body myopathy [174,177]	sporadic	Late adult	Quadriceps and/or finger flexors	Dysphagia, respiratory involvement	Progressive, LoA in later stages	Very variable, ranging from normal to up to 15 times UNL	Rimmed vacuoles and protein aggregates ++
oculo-pharyngeal muscular dystrophy [186,191]	*PABPN1* */AD	Late adult	Eyelid ptosis+ bulbar	Dysphagia, respiratory involvement, executive function deficits, rarely peripheral neuropathy	Slowly progressive	Slightly elevated in mild cases, higher severe cases	Rimmed vacuoles +/−
FHL1 myopathy [180]	*FHL1* ^†^/XLD	Young adult	Neck flexors and limb-girdle	Respiratory involvement, scoliosis, rigid spinel, rarely cardiomyopathy	Severe progressive course, LoA at early stages	Mildly elevated	Protein aggregates +
PLIN4 myopathy [8,189,190]	*PLIN4* §/AD	Adult	Distal and limb-girdle	None	Slowly progressive, LoA at early stages	Normal to slightly elevated	Rimmed vacuoles and preotein aggregates +

AD: autosomal dominant, AR: autosomal recessive, XLD: X-linked dominant LoA: loss of ambulation, UNL: upper normal limit. * Abnormal expansion of (GCN)n repeats, ^†^ p.C150R, § 99 nt coding expansion.

## 4. Conclusions

Aggrephagy represents an underexplored but widely-present pathological mechanism in the more general context of autophagy, with the latter already being extensively investigated in human diseases, including myopathies. To date, aggrephagy has been investigated only in a limited number of diseases, reveling a pathological role in a case of *FHL1*-rigid spine syndrome and in a small case series of sIBM, *PLIN4*-related myopathy, myotilinopathies and desminopathies and oculopharyngeal muscular dystrophy.

Notably, these diseases are extremely heterogeneous. The described myopathies are all genetically defined, apart from sIBM, which is an acquired entity, suggesting a transversal role of aggrephagy across different diseases. The clinical presentation mainly occurs in adult life, around the fifth and sixth decades, and shows a slowly progressive worsening, with the exception of RSS cases, which have an onset in early adult life and have a rapid decrease of motor functions. These observations suggest that the impairment of aggrephagy is generally a slowly ongoing progressive process that may be related to aging. On the other hand, the distribution of affected muscles is very diverse, ranging from cranial to limb-girdle to distal muscle involvement. Even cardiomyocytes can be affected, as in desminopathy, myotilinopathy [192] and *FHL1*-related disorders. Accumulation of protein aggregates implicating the aggrephagy pathway abnormality has been recently investigated in patients with genetic cardiomyopathy. The analysis of p62-positive aggregates in the myocardium showed a high prevalence of diffused p62-positive aggregates in phospholamban (PNL) and desmin-related (DRC) cardiomyopathy patients [193], confirming earlier findings in a mouse model of DRC desmin mutant and impaired lysosomal function [194].

Thus far, only a few players known to be directly involved in the selective removal of protein aggregates have been investigated in muscle diseases. Most likely, the fact that p62 and NBR1 appear to be more investigated in these diseases compared to the other known aggrephagy players is related to a better knowledge of their function and mechanism of action. However, the simultaneous evaluation of other proteins involved in aggrephagy is necessary for a comprehensive view of the possible pathogenic mechanism leading to protein aggregation myopathies.

Further studies are needed to better characterize the pathological role of aggrephagy in muscle diseases and to define possible therapeutic targets.

## Data Availability

Not applicable.
